# Quality of Life Assessment in Women With Breast Cancer in Nineveh, Iraq

**DOI:** 10.7759/cureus.51589

**Published:** 2024-01-03

**Authors:** Israa J Mustafa, Omar R Abdullah, Najlaa Al-Saffar, Rashad F Ahmed, Mohammad Fouad

**Affiliations:** 1 Cancer Control Center, Oncology and Nuclear Medicine Hospital, Mosul, IRQ; 2 Department of Nursing, Al-Hadba'a University College, Mosul, IRQ; 3 Department of Anesthesiology, Al-Hadba'a University College, Mosul, IRQ; 4 College of Medicine, University of Mosul, Mosul, IRQ

**Keywords:** follow-up, chemotherapy, fact-b, quality of life, breast cancer

## Abstract

Background

Due to earlier detection and improved treatment and examinations, there has been a rise in the survival rate of patients with breast cancer (BC). It is imperative to examine the health-related quality of life (QoL) of these patients, as it can aid healthcare professionals and authorities in comprehending the variables that influence quality of life. Unfortunately, there is a dearth of information on the subject of women in Nineveh. In Nineveh, a study sought to delve into the quality of life experienced by women undergoing treatment or on follow-up for breast cancer at Mosul Oncology and Nuclear Medicine Hospital. Specifically, researchers wanted to see how age and treatment impacted the QoL of these women.

Methods

A cross-sectional study was conducted on 212 women with BC. Clinico-pathological-, social-, disease-, and treatment-related characteristics were reviewed. The Functional Assessment of Cancer Therapy-Breast (FACT-B) questionnaire was used for assessing QoL in this study.

Results

The overall FACT-B score was 75.7 (SD=19.99). Around 36.3% (n=77) of women with BC suffered from a lack of energy, and 39% (n=83) could not meet the needs of their families. The mean score of emotional well-being was the lowest among the FACT-B subscales.

Patients aged 60 years and older had significantly worse QoL than younger patients (less than 60 years), and patients receiving chemotherapy had poor QoL. During the chemotherapy period, 51.4% (n=77) of patients were bothered by the side effects of treatment; 43.8% (n=65) suffered from pain; 35.3% (n=53) had nausea; and 39.1% (n=58) felt ill.

Conclusions

Patients with breast carcinoma who are older tend to experience a lower quality of life, according to the findings of a recent study. Interestingly, those who undergo systemic chemotherapy have a worse QoL than their counterparts who complete their chemotherapy cycles. As a result, healthcare providers must offer targeted interventions to improve quality of life, particularly for those who fall into the older age group and those receiving chemotherapy.

## Introduction

Breast cancer (BC) reigns as the most widespread form of cancer afflicting women across the globe, as per the latest estimates by the WHO, which covered 185 nations. Shockingly, over a hundred nations record it as the primary cause of cancer deaths. Iraq, too, is grappling with this menace, with it being the most prevalent among the ten major cancers in the country. Females account for 4922 out of 4996 cases, making up 98.5% of all cancer cases [1].

Different modalities are employed for treating BC patients, including surgery, chemotherapy, radiation, hormonal therapy, and immunotherapy. To best approach BC treatment, patients usually require a customized combination of these modalities dependent on their BC stage and type. In addition to the physical distress caused by these procedures, emotional stress is also a common outcome due to side effects such as anxiety, social devaluation fears, and a preoccupation with mortality [2].

In recent observations, the survival rate for patients with BC has increased, prompting clinicians to investigate the effects of treatment on patients' health-related quality of life (QoL) [3]. Patients undergoing treatment for BC may experience a range of negative impacts on their psychological well-being, including functional inability, altered self-image, loss of breast sensitivity, anxiety, depression, and other emotional and psychological changes [4]. Treatment complications and side effects have a detrimental effect on the QoL of those with BC [1]. Numerous studies have demonstrated that QoL indicators deteriorate following a diagnosis of BC, specifically in terms of breast cancer [2,5]. During and after treatment, it is important to evaluate a patient's quality of life (QoL) to make informed decisions about how to alleviate their suffering. By meeting the needs of cancer patients, we can help them live healthier lives. This highlights the necessity of addressing QoL issues [6].

Breast cancer patients' quality of life (QoL) is determined by a variety of factors, such as diagnosis, treatment, daily activities, and the recovery process [7]. The assessment of QoL can be done using different types of tools that come in various forms. FACT-B is a questionnaire used as one of the tools in evaluating breast cancer patients' QoL [8]. In assessing QoL for BC treatment, FACT-B (Version 4) was found effective with reliability and validity. The sample of 280 participants proved its ease of administration, brevity, and sensitivity to change. This was demonstrated alongside good results in eight areas [8,9].

There is a lack of data about the QoL of BC patients in Nineveh. Therefore, we found that there is a demand to explore the QoL of patients with BC and what their main concerns are. In Nineveh, health institutions and infrastructure were severely affected by the last war against the Islamic State of Iraq and the Levant (ISIL), which reflected badly on cancer patients’ management. Patients with cancer could be one of the most affected groups, as they require advanced centers and expensive therapies. Thus, QoL during and after the treatment period might be negatively affected [10].

The present study was aimed at exploring the QoL of women with BC who were under treatment or on follow-up at the Oncology and Nuclear Medicine Hospital, Mosul, a tertiary cancer hospital in Nineveh.

## Materials and methods

In Nineveh Governorate, Iraq, a cross-sectional study was conducted at the Oncology and Nuclear Medicine Hospital, Mosul to attain the research goals.

Following a review of the registered medical files by the records team, patient identification commenced, and only those who met the inclusion criteria could be enrolled. Once found eligible, a learned assistant notified and educated the patients regarding the study at hand. Out of the 212 women diagnosed with breast cancer, they all freely consented to participate in this current study. Upon receiving consent, the research assistant proceeded to conduct a face-to-face interview with each individual to assist in questionnaire completion.

The collection of data was achieved through a structured questionnaire with two distinguishable parts, specifically designed to elicit key information about the disease, types of treatment, and the socio-demographic characteristics of patients. Additionally, a specialized questionnaire aimed at assessing the quality of life (QoL) specifically for breast cancer patients was employed, known as the Functional Assessment of Cancer Therapy-Breast (FACT-B) questionnaire, Version 4. This useful instrument not only focuses on the physical aspect but also explores emotional, social, and deeper concerns commonly faced by cancer patients. As a standardized tool, the FACT-B questionnaire was thoughtfully translated into the Arabic language to cater to the patient's language preference.

With 37 questions, the FACT-B tool has two distinct sections. The first, FACT-General (FACT-G), assesses overall health and consists of four components: physical well-being (PWB; seven questions), social/family well-being (SWB; seven questions), emotional well-being (EWB; six questions), and functional well-being (FWB; seven questions). Additionally, the Breast Cancer Specific (BCS) domain features 10 questions relating to the impact of cancer and its treatment. These questions delve into the physical, aesthetic, and psychological disorders arising from the illness [11]. Of all subscales, the maximal score for FACT-B-Total is 148, indicating an ideal quality of life. The scale for every question, on the other hand, ranges from 4 (highest) to 0 (lowest) [12].

Calculating the Trial Outcome Index (TOI) can reveal notable changes in both physical and functional outcomes. Rather than relying on a multidimensional score, TOI can be an alternative method for gauging adjustments in these areas. The sum of PWB, FWB, and BCS can also be used to compute the TOI [13].

The inclusion criteria included female patients aged eighteen years old or older with histologically proven breast cancer who were treated at Mosul Oncology and Nuclear Medicine Hospital in 2022. While patients with other malignancies or in critical condition were excluded. A comparison was made between patients under chemotherapy vs. follow-up and age (<60 vs. ≥60).

Analysis

Participants' general and clinical characteristics were presented using a descriptive analysis at the onset.

Life's standard was evaluated using PWB, SWB, EWB, FWB, BCS, FACT-B-TOI, FACT-G-Total, and FACT-B-Total components. The mean and standard deviation for each FACT-B subscale were pitted against patients on chemotherapy and follow-up, as well as age groups (<60 years vs. ≥60 years) using analysis of variance (ANOVA) and an independent sample t-test. Significance was attributed to P-values≤0.05.

Ethical approval

Agreement with the ethical principles of the Declaration of Helsinki was reached for this study. On October 25, 2022, the Ethics Committee of the Training and Human Development Center of Nineveh Health Directorate approved the research project. Following a brief clarification of study objectives and the provision of guarantees regarding privacy, confidentiality, and data security, each participant provided written informed consent to the research assistant.

## Results

From January 2022 to January 2023, a total of 245 patients with BC were approached, and only 212 patients were recruited at the Oncology and Nuclear Medicine Hospital in Mosul, Iraq. All 212 patients completed the amended FACT-B questionnaires with the support of the research team, and their data were analyzed.

Demographic, disease and treatment-related characteristics of study participants

In this sample, 70.7% were <60 years of age (n=150), 89% (n=189) were housewives, 6% (n=13) had a job and were (n=124) working, 73.3% (n=155) were married, 68.5% (n=145) were free from co-existing health issues, and nearly 58.7% were postmenopausal. Around 92% (n=195) and 91% (n=193) of women had an initial mastectomy and received adjuvant chemotherapy, respectively. Of the patients, 50% (n=106) received radiation therapy, and 27% (n=57) received radiation therapy after finishing adjuvant chemotherapy. The disease-related characteristics of participants are listed in Table 1.

**Table 1:** Disease- and treatment-related characteristics of the study population (n=212) HER2: human epidermal growth factor receptor 2; P(N): number of lymph nodes involved

Characteristics	Number	%
Side of breast cancer		
Bilateral	5	2.3
Left breast	106	50
Right breast	101	47.6
Stage at diagnosis		
In situ	3	1.6
Localized	182	85.8
Metastatic	25	11.7
Progesterone receptor		
Positive	133	62.7
Negative	79	37.2
Estrogen receptor		
Positive	167	78.7
Negative	45	21.2
HER2		
Positive	63	29.7
Negative	149	70.2
Ki-67		
Negative	80	37.6
Positive	68	32.1
Unknown	64	30.3
Tumour size		
≤2 cm	41	19.3
2-5 cm	123	58.0
>5 cm	38	17.9
Unknown	10	0.4
Number of lymph nodes involved		
No L.N. (pN0)	57	26.9
1-3 (pN1)	66	31.1
4-9 (pN2)	36	16.9
>10 (pN3)	5	2.3
Not sure (pNX)	48	22.5

Quality of Life

Table 2 displays the FACT-B response distribution. PWB, SWB, EWB, FWB, BCS, FACT-B-TOI, FACT-G-Total, and FACT-B-Total variables were all measured and displayed with their mean scores and standard distributions through the FACT-B scores obtained.

**Table 2:** Mean and standard deviation values for all functional assessments in breast cancer patients SD: standard deviation

Variable	Mean± SD	Score range
Physical well-being	11.9±5.33	0-28
Social well-being	18.7±4.53	0-28
Emotional well-being	9.7±3.40	0-24
Functional well-being	13.7±5.48	0-28
Breast cancer-specific concerns	21.7±4.59	0-40
Trial Outcome Index	47.3±13.95	0-96
Functional Assessment of Cancer Therapy-General	54.0±15.43	0-108
Functional Assessment of Cancer Therapy-Breast	75.7±19.99	0-148

According to the items of physical well-being, about 36.3% (n=77) of women with BC cancer suffered from a lack of energy, and 35.3% (n=75) of all had severe nausea. About 39.1% (n=83) could not meet the needs of their family.

Of the patients, 43.8% (n=65) suffered from pain due to different causes, especially during the chemotherapy period. The side effects of the treatment bothered them. Around 51.4% (n=77) and 39.1% (n=58) felt ill, and 54.2% (n=81) of patients were forced to spend time in bed. The mean score of emotional well-being was the lowest among the FACT-B subscales.

Comparison of quality of life according to the age group and time of questionnaire administration to the patients with breast cancer

To pinpoint prime concerns, an examination was conducted on each item within the subscale. The mean assessments of EWB and BCS highlighted no noticeable disparities between the two age groups. However, those younger than 60 years old boasted superior standards of living compared to their counterparts over 60 years old. Most notably, significant contrasts surfaced in the mean scoring for PWB, SWB, FWB, TOI, FACT-G, and FACT-B.

However, the quality of life according to the time of questionnaire administration to the patients showed that only the mean scores of PWB and FACT-G displayed a significant difference between the mean scores of patients with breast cancer during and after receiving chemotherapy. Patients receiving chemotherapy have a lower quality of life, according to the FACT-B subscales. All items in physical well-being for patients under chemotherapy were significantly lower than for patients under follow-up.

There are significant sociodemographic and clinical differences in this population by age category. Compared with women aged ≥60 years, women aged <60 years had higher education levels (p<0.01), higher employment rates (p<0.001), and significantly higher levels of daily activities (p<0.05).

As shown in Table 3, the results of the t-test indicate that age has a great impact on quality of life. The quality of life of people over 60 years old is significantly lower than that of younger people. Patients aged 60 years and older had the lowest quality of life scores (FACT-B) of all subgroups. To investigate which subscale of physical health is associated with age, we compared the means of the respective FACT-PWB items between the two age groups. However, not all FACT-PWB items scored lower at older ages. Younger women showed significantly worse outcomes in GP5 (p<0.02), GP6 (p<0.01), and GP7 (p<0.001). This means women under 60 are more likely to experience treatment side effects, have more severe disease, and spend more time in bed than older women. Since higher scores indicate a better quality of life, patients who participated in follow-up visits had the best overall quality of life.

**Table 3:** Comparison of quality of life according to the age group and time of questionnaire administration to patients with breast cancer SD: standard deviation; PWB: physical well-being; SWB: social/family well-being; EWB: emotional well-being; FWB: functional well-being; BCS: breast cancer-specific issues; FACT-B: Functional Assessment of Cancer Therapy-Breast; TOI: Trial Outcome Index; FACT-G-Total: Functional Assessment of Cancer Therapy-General-Total *A license to use this module and assessment system can be purchased at www.facit.org. The Friedman test was used to determine repeated measures differences. Overall, the median value of each variable over time was considered.

Variable	Age		Sig	Time		Sig	
	<60 years	≥60 years		On chemotherapy	Post chemotherapy		
	(n=150)	(n=62)		(n=149)	(n=63)		
	Mean± SD	Mean± SD		Mean± SD	Mean± SD		
PWB	12.55±3.23	10.45±3.88	0.050	11.06±3.15	14.00±3.23	0.00	
SWB	19.51±3.72	16.95±2.78	0.002	18.68±2.80	18.95±2.86	0.74	
EWB	8.25±1.95	9.17±2.93	0.258	8.34±2.49	8.95±2.20	0.45	
FWB	14.68±4.50	11.41±3.19	<0.001	12.30±3.37	13.73±3.69	0.14	
BCS	21.75±7.01	21.82±5.49	0.945	21.87±8.78	21.54±4.38	0.73	
TOI	48.98±10.46	43.68±10.33	0.028	45.23±9.77	49.27±11.71	0.09	
FACT-G	54.99±16.56	47.98±9.41	0.008	50.38±14.40	55.63±13.8	0.04	
FACR-B	76.74±20.67	69.82±14.11	0.037	72.45±16.74	77.17±18.52	0.13	

## Discussion

In this study, the mean FACT-B score for all patients was 75.7 (SD=19.99), which is within the global range. Globally, the average reported overall quality of life score ranges from 57.23 to 84.39, depending on the questionnaire used in the included studies [6].

The results of the current study showed that women with BC had the highest mean score for social well-being at 18.7 (SD=4.53), followed by functional well-being at 13.7 (SD=5.48), physical well-being at 11.9 (SD=5.33), and emotional well-being at 9.7 (SD=3.40). As a result, physical and emotional well-being scores are the lowest and significantly impact quality of life. When BC is diagnosed, patients often experience extreme anxiety, a sense of danger and unease, and often suffer from depression. These reactions are due to society's belief that cancer is a distressing disease that is ultimately fatal [2,7].

This study found that breast cancer survivors experienced more physical and emotional distress during chemotherapy. The increased risk comes from receiving chemotherapy, which can cause various complications, and most patients experience ups and downs during treatment. Likewise, numerous studies have demonstrated that chemotherapy has an impact on the quality of life of BC patients [2]. Women undergoing chemotherapy require more support, so most of them find that they are unable to lead a normal life during chemotherapy. After chemotherapy, they found daily life more difficult, but they recovered quickly during treatment. Recognizing these specific physical and psychological consequences can help healthcare professionals specifically identify possible effects that require special attention. Healthcare providers should continue to treat systemic symptoms in BC patients receiving chemotherapy. However, we found that women with follow-up had the best overall quality of life, suggesting that quality of life improved after the completion of systemic chemotherapy.

Our study shows that older women with BC have a significantly worse quality of life. Therefore, various interventions should be provided to elderly breast cancer patients to improve their quality of life. Data analysis shows that patients under 60 years old have better PWB effects than older patients. Due to the aging process, patients aged 60 years and older have more difficulty managing their physical activity than younger groups [14]. However, the younger group experienced more treatment side effects, a more severe illness, and more time in bed than the older group. Therefore, it is important to provide good support to patients undergoing chemotherapy.

Quality of life assessment is an important parameter in assessing how survivors cope with the impact of the disease and its treatment. Numerous studies have shown that BC survivors suffer from numerous adjustment problems and require rehabilitation and support services [12,13]. Quality of life information can help health planners identify patient services that should be maintained or developed.

It is known that socioeconomic status and education are significantly associated with quality of life and depression [15]. Women under 60 are more likely to have higher levels of education and more frequent employment, which contributes to better access to information and problem-solving resources and better coping skills, which may explain a better quality of life. Providing education and information about BC, its management, and associated resources can help improve the quality of life of older patients.

BC causes a significant deterioration in a patient's quality of life, which is exacerbated by a lack of information and appropriate rehabilitation options. In Nineveh, deterioration in cancer care is one of the leading causes of reduced quality of life for BC women due to the impact of the recent conflict on the province's health system. In Nineveh, fragmented private services are increasingly filling cancer care gaps caused by the war. Additionally, Nineveh lacks public oncology facilities such as radiotherapy machines, pain clinics, and palliative care clinics. These services are limited to the basic diagnosis and administration of chemotherapy without functional radiation therapy or positron emission tomography (PET scan) [10]. Additionally, Nineveh does not have sentinel lymph node biopsy (SLNB), which can reduce the risk of side effects of axillary lymph node dissection, such as pain, numbness, lymphedema, and arm and shoulder stiffness.

To improve cancer care and the quality of life of cancer patients, it is important to equip public institutions with the capacity to deliver better, more comprehensive care. Proper counseling to patients and families regarding anxiety, depression, sexual problems, diet, and exercise is highly recommended to improve the quality of life of patients with BC.

Incorporating quality of life assessment into clinical practice for women with BC has great potential to benefit patients [16]. BC patients need effective communication with their healthcare team and resolution of their primary condition. Additionally, patients need to be encouraged to acknowledge and express their feelings. Health professionals need to evaluate patients' quality of life issues in detail and provide effective treatment for physical problems such as pain, limb swelling, and fatigue.

When BC is diagnosed, patients often experience severe anxiety, a sense of imminent danger, uneasiness, and often sadness. These reactions are due to the public's perception that cancer is a terrible disease that is ultimately fatal.

Limitations

In order to thoroughly explore the variations in QoL levels and influential factors in Iraqi BC patients, additional research needs to be conducted, and a suitable questionnaire should be identified that accurately reflects QoL in BC patients. FACT-B, in particular, proved to be challenging for participants as they were unfamiliar with the standard of care they should be receiving, thus hindering their ability to answer many of the questions. Mixing patients with varying stages and treatments also posed a potential limitation. Future studies should aim to define patients' stages and treatment characteristics in order to obtain more comprehensive data about QoL.

## Conclusions

For patients undergoing chemotherapy and those reaching an advanced age, their quality of life is significantly impacted by physical and psychological symptoms as well as their sense of belonging. Thus, it's crucial to provide interventions tailored toward enhancing the quality of life for those facing breast cancer. Notably, the quality of life has greatly improved for patients who have completed their chemotherapy sessions successfully.

The provision of restricted oncology healthcare services may have had a significant impact on the quality of life of BC patients.
